# Exchange Bias Modulated by Antiferromagnetic Spin‐Flop Transition in 2D Van der Waals Heterostructures

**DOI:** 10.1002/advs.202307034

**Published:** 2024-02-14

**Authors:** Kai Gu, Xiaoqian Zhang, Xiangjie Liu, Xinlei Guo, Zhenqi Wu, Shuo Wang, Qinxin Song, Wei Wang, Lujun Wei, Ping Liu, Jingrui Ma, Yongbing Xu, Wei Niu, Yong Pu

**Affiliations:** ^1^ New Energy Technology Engineering Laboratory of Jiangsu Province & School of Science Nanjing University of Posts and Telecommunications Nanjing 210023 China; ^2^ Key Laboratory of Quantum Materials and Devices of Ministry of Education School of Physics Southeast University Nanjing 211189 China; ^3^ International Quantum Academy Shenzhen 518048 China; ^4^ Key Laboratory of Flexible Electronics & Institute of Advanced Materials Jiangsu National Synergetic Innovation Center for Advanced Materials Nanjing Tech University Nanjing 211816 China; ^5^ Key Laboratory of Energy Conversion and Storage Technologies Southern University of Science and Technology Shenzhen 518055 China; ^6^ School of Electronic Science and Engineering Nanjing University Nanjing 210023 China

**Keywords:** 2D magnet, exchange bias, spin‐flop transition, van der Waals heterostructure

## Abstract

Exchange bias is extensively studied and widely utilized in spintronic devices, such as spin valves and magnetic tunnel junctions. 2D van der Waals (vdW) magnets, with high‐quality interfaces in heterostructures, provide an excellent platform for investigating the exchange bias effect. To date, intrinsic modulation of exchange bias, for instance, via precise manipulation of the magnetic phases of the antiferromagnetic layer, is yet to be fully reached, owing partly to the large exchange fields of traditional bulk antiferromagnets. Herein, motivated by the low‐field spin‐flop transition of a 2D antiferromagnet, CrPS_4_, exchange bias is explored by modulating the antiferromagnetic spin‐flop phase transition in all‐vdW magnetic heterostructures. The results demonstrate that undergoing the spin‐flop transition during the field cooling process, the A‐type antiferromagnetic ground state of CrPS_4_ turns into a canted antiferromagnetic one, therefore, it reduces the interfacial magnetic coupling and suppresses the exchange bias. Via conducting different cooling fields, one can select the exchange bias effect switching among the “ON”, “depressed”, and “OFF” states determined by the spin flop of CrPS_4_. This work provides an approach to intrinsically modulate the exchange bias in all‐vdW heterostructures and paves new avenues to design and manipulate 2D spintronic devices.

## Graphical abstract

Exchange bias in the all‐vdW magnetic heterostructure is modulated by the antiferromagnetic spin‐flop phase transition of CrPS_4_. Via the spin flop, one can select the exchange bias switching among the “ON”, “depressed”, and “OFF” states. This work provides an approach to intrinsically modulate the exchange bias in all‐vdW heterostructures and paves avenues to design and manipulate 2D spintronic devices.

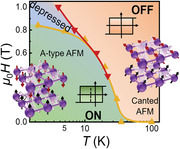

## 1 Introduction

With the emergence and extensive research on 2D van der Waals (vdW) magnetic materials,^[^ ^1^, ^2^ ^]^ heterostructures comprising vdW magnets exhibit a particularly rich variety of novel phenomena, such as chiral spin textures,^[^ ^3^, ^4^ ^]^ magnetic proximity effect,^[^ ^5^, ^6^ ^]^ quantum anomalous Hall effect,^[^ ^7^ ^]^ exchange bias effect,^[^ ^8^ ^]^ etc. In particular, in the vdW magnets constituted ferromagnet/antiferromagnet (FM/AFM) heterostructures, perfect interfaces without dangling bonds or interfacial defects, are capable of investigating the interface‐related effect, especially for the exchange bias effect. Moreover, interfacial magnetic interactions play a prominent role in vdW heterostructures due to the weak interlayer coupling of the vdW magnets. Therefore, exchange bias is highly expected in vdW FM/AFM heterostructures and its underlying mechanism can be well explored.

Indeed, with the rapid increase of the types of vdW magnets, the exchange bias effect has been widely observed in various 2D vdW FM/AFM heterostructures, for instance, Fe_3_GeTe_2_ (FGT)/MnPX_3_ (X = S and Se),^[^ ^5^, ^9^, ^10^ ^]^ FGT/FePX_3_ (X = S and Se),^[^ ^11^, ^12^ ^]^ FGT/CrCl_3_,^[^ ^13^ ^]^ FGT/Molecular,^[^ ^14^ ^]^ FGT/CrOCl,^[^ ^15^ ^]^ FGT/Oxidized‐FGT,^[^ ^16^, ^17^ ^]^ Fe_5_GeTe_2_/FePS_3_,^[^ ^18^ ^]^ Cr_2_Ge_2_Te_6_/MnBi_2_Te_4_,^[^ ^19^ ^]^ and CrI_3_/MnBi_2_Te_4_.^[^ ^20^ ^]^ It is obvious that FGT and its series are generally adopted as the FM layer, since their high Curie temperature (*T*_C_) and strong perpendicular magnetic anisotropy (PMA).^[^ ^21^, ^22^ ^]^ Among various vdW AFM materials, CrPS_4_ (CPS), with a ground state of A‐type AFM below the Néel temperature (*T*_N_) of 38 K,^[^ ^23^, ^24^, ^25^ ^]^ has uncompensated magnetic spins in a single layer. This uncompensated layer at the interface meets the requirement of the exchange bias to pin the magnetic moments. More importantly, with relatively low magnetic fields, CPS could achieve magnetic phase transitions of a spin flop (<1 T) and a spin flip (∼8 T), due to the much weaker interlayer exchange coupling of 2D vdW CPS compared with conventional bulk AFM.^[^ ^24^ ^]^ While some recent studies have investigated the properties of CPS,^[^ ^23^, ^24^, ^25^, ^26^ ^]^ the exchange bias of all‐vdW heterostructures involving the CPS as the AFM layer has yet to be reached. Thus, the vdW heterostructure of FGT/CPS provides an ideal platform to investigate the exchange bias.

For spintronic devices based on exchange bias, great efforts are paid to optimize the strength of exchange bias field (*H*_ex_). Besides, effective modulation of exchange bias is also urgently necessary for practical applications. Recently, some external methods, such as proton intercalation,^[^ ^18^, ^27^ ^]^ gate voltage,^[^ ^19^ ^]^ pressure engineering,^[^ ^12^, ^28^ ^]^ and surface oxidization,^[^ ^16^, ^17^, ^29^ ^]^ have been conducted to tune the exchange bias effect in 2D vdW heterostructures. Although these extrinsic stimuli have enriched the application scenario of exchange bias, intrinsic modulation of exchange bias, for instance, via the manipulation of the precise magnetic phases of the AFM layer, is still in demand for integration. Inspired by the easily accessible spin‐flop transition between A‐type AFM state and canted AFM one of CPS at relatively low fields, the concern of whether the spin‐flop transition could modulate the exchange bias is intriguing and worthwhile.

Herein, the exchange bias effect has been observed in both FGT/CPS and (Fe_0.74_Co_0.26_)_3_GeTe_2_ (FCGT)/CPS, archetypes of all‐vdW FM/AFM heterostructures. Negative exchange bias is evidenced in both heterostructures due to the ferromagnetic coupling at the interfaces. Proper cooling field (*H*_CF_) and thickness of FGT are required for FGT/CPS heterostructures to gain the expected exchange bias. The depressed coercivity (*H*_C_) of FCGT via doping, smaller than the spin‐flop field (*H*_SF_) of CPS, is capable of achieving the effective modification of the exchange bias in FCGT/CPS by the spin‐flop transition. Adopting a *H*_CF_ surpassing the *H*_SF_, CPS turns from A‐type AFM to canted AFM, thus the exchange bias effect is depressed or even eliminated. In this way, we can select the desired states of exchange bias by an intrinsic approach of the convenient field‐cooling process. This work offers a deep understanding of the spin‐flop‐modulated exchange bias in all‐vdW heterostructures. This understanding facilitates the design and manipulation of spintronic devices on the basis of 2D vdW magnets.

## 2 Results and Discussion

**Figure 1:**
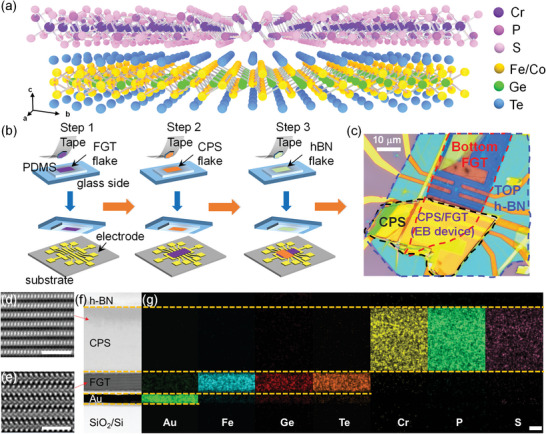
Basic characterization of 2D vdW FM/AFM heterostructures. a) Schematic diagram of the FGT (FCGT)/CPS magnetic heterostructure. b) Fabrication process of the FGT (FCGT)/CPS nanodevice. c) Optical image of the FGT/CPS vdW heterostructure. d,e) HADDF‐STEM images of CPS and FGT, respectively. The scale bar is 2 nm. f,g) Cross‐sectional ABF‐STEM image and corresponding EDS mappings of the FGT/CPS heterostructure encapsulated by *h*‐BN on the SiO_2_/Si substrate with pre‐deposited Au electrode. The scale bar is 20 nm.

**Figure** **1**a illustrates the vdW heterostructure of FGT/CPS (or FCGT/CPS), where vdW gaps without dangling bonds provide a high‐quality interface. Different from the cases of traditional exchange bias effect in thin‐film heterostructures, where interfacial defects and diffusions normally play a pronounced role, perfect interfaces among vdW heterostructures allow us to investigate the intrinsic magnetic coupling mechanisms of the exchange bias effect. FGT/CPS (and FCGT/CPS) vdW heterostructures were fabricated as depicted in Figure 1b. FGT (FCGT) flakes with different thicknesses (11.8–52 nm) were mechanically exfoliated from the bulk crystal and transferred onto electrodes using a dry transfer method with polydimethylsiloxane (PDMS), and the CPS flake (the thickness range of CPS is 10.7–148 nm. Note that the thickness of most CPS flakes we chose is ≈120 nm due to its easy accessibility and sufficient pinning of antiferromagnetism) was then transferred onto the FGT flakes, followed by encapsulation of the entire heterostructure with *h*‐BN to prevent oxidation and contamination. Recently, the exchange bias has been observed in FGT with surface oxidization,^[^ ^16^ ^]^ however, the intrinsic magnetic couplings between FGT and CPS are more desired in this work. In order to distinguish the genuine exchange bias of FGT/CPS from the oxidized‐FGT/FGT (Figure S3, Supporting Information), the device (with two standard 6‐electrode Hall‐bar configurations) of a large‐area FGT (52 nm of the thickness) partially covered by CPS, as shown in Figure 1c of the optical image, was fabricated to measure the individual FGT and the heterostructure, separately. Moreover, the cross‐sectional scanning transmission electron microscope (STEM) images and corresponding energy‐dispersive X‐ray spectroscopy (EDS) mappings were employed to further validate the quality of the FGT/CPS heterostructure. The annular bright‐field (ABF)‐STEM image (Figure 1f) gives a picture of the FGT/CPS heterostructure encapsulated by *h*‐BN on the SiO_2_/Si substrate with pre‐deposited Au electrode, displaying flat and homogeneous interfaces. High‐resolution high‐angle annular dark field (HAADF)‐STEM images (Figure 1d,e) show the high‐quality crystal structures of CPS and FGT, respectively. Moreover, the EDS mappings demonstrate a clear distribution of different elements in corresponding regions, further signifying the clean and homogeneous interface of the FGT/CPS heterostructure.

**Figure 2:**
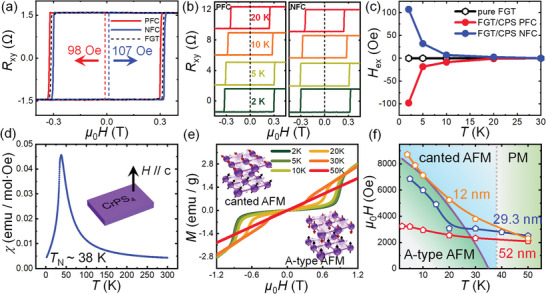
Exchange bias effect in FGT(52 nm)/CPS(120.2 nm) heterostructure and the magnetic properties of CPS. a) Typical magnetic field dependence of Hall resistance, *R*_xy_, measured at 2 K for FGT(52 nm)/CPS(120.2 nm) under both PFC and NFC and the individual FGT without field cooling. b) Magnetic field dependence of *R*_xy_ of FGT(52 nm)/CPS(120.2 nm) at various temperatures under a *H*_CF_ of 0.5 T adopted in positive and negative fields, respectively. c) Temperature‐dependent *H*_ex_ of FGT(52 nm)/CPS(120.2 nm). d) Magnetic susceptibility of CPS as a function of the temperature, showing a *T*_N_ of ≈38 K. The magnetic field of 1000 Oe is applied along the *c‐*axis. e) Field dependence of the magnetization of CPS at various temperatures with *H*//*c*‐axis. The insets schematically show the spin textures of the A‐type AFM state and the canted‐AFM one, respectively. f) Magnetic phase diagram of CPS and the temperature dependence of *H*_C_ of FGT with different thicknesses. The purple solid line indicates *H*_SF_ of CPS at different temperatures.

To explore the exchange bias in FGT/CPS heterostructure, magnetotransport measurements are employed to record the Hall resistance (*R*_xy_) with the anomalous Hall effect (AHE). Hall signal of the individual FGT region was initially measured without field cooling, then standard measurement methodology of the exchange bias effect with field cooling was conducted on the heterostructure. As shown in **Figure** **2**a, at a typical temperature of 2 K, Hall resistance of the pure FGT region (black dashed line) is oddly symmetric relative to the zero point, manifesting no exchange bias in pure FGT and further excluding the possibility of the oxidization induced exchange bias in our samples. More transport results of individual FGT region can be referred to in Figure S2 (Supporting Information). In contrast, for the FGT(52 nm)/CPS(120.2 nm) heterostructure, obvious lateral shifts of the AHE loop are observed under both the negative field cooling (NFC) of −0.5 T and positive field cooling (PFC) of +0.5 T. In the case of NFC, the shift is toward the positive magnetic field, while the opposite shift is toward the negative one for the PFC. This indicates a negative exchange bias exhibits at the interface of FGT/CPS, which is typically found in most vdW magnetic heterostructures.^[^ ^11^, ^13^, ^14^ ^]^

Figure 2b summarizes Hall resistance at different temperatures under a PFC and an NFC process, respectively. It is seen that the shifts degrade as increasing the temperature. Exchange bias field, *H*_ex_, is defined as *H*_ex_ = (*H*_C_^+^ + *H*_C_^−^)/2, where *H*_C_^+^ and *H*_C_^−^ are the positive and negative coercivity, respectively. Figure 2c shows the temperature dependence of *H*_ex_ from 2 to 30 K. The magnitude of these *H*_ex_ induced by the PFC and NFC is identical within experimental error and decreases with increasing the temperature. The exchange bias effect disappears at 20 K, signifying a blocking temperature (*T*_B_) for this FGT(52 nm)/CPS(120.2 nm) heterostructure.

To deeply understand the magnetic couplings of this vdW heterostructure, the magnetic properties of the antiferromagnetic CPS are investigated. Figure 2d shows the temperature‐dependent susceptibility of the CPS. Upon cooling, the susceptibility initially increases and then decreases sharply, forming a predominant peak in this curve. *T*_N_ is manifested to be ≈38 K as defined from the peak of this magnetic susceptibility, consistent with previous studies.^[^ ^24^, ^25^ ^]^ Magnetization as a function of the applied out‐of‐plane field including the backward‐forward sweeps at different temperatures is displayed in Figure 2e. It should be noted that the backward‐ and forward‐sweeps are fully overlapped, distinct from the cases of monolayer or odd‐layer CrI_3_ or CPS.^[^ ^30^, ^31^ ^]^ For temperatures below *T*_N_, magnetizations increase faintly as the field increases in the low magnetic field region, which is a typical AFM feature. Distinctively, increasing the magnetic field to a critical one (namely *H*_SF_), magnetizations increase sharply and nonlinearly, which is the field‐driven spin‐flop phase transition.^[^ ^24^ ^]^ Undergoing this phase transition, the A‐type AFM ground state of CPS transforms into a canted AFM one, of which magnetic moments are aligned antiferromagnetically perpendicular to the applied field but with small net moments along the easy axis. The insets of Figure 2e illustrate the alignments of magnetic moments before and after the spin‐flop transition. The *H*_SF_ decays with increasing temperature and the spin‐flop transition vanishes when the temperature is higher than 35 K. Figure 2f summarizes the magnetic phases diagram of CPS under different temperatures and applied magnetic fields. When the temperature is below *T*_N_, CPS turns from the paramagnetic (PM) phase to the AFM one. In a low field region below *T*_N_, CPS is in its ground state of A‐type AFM. When the magnetic field surpasses the *H*_SF_ (the solid purple line in Figure 2f), the spin‐flop transition begins and the CPS becomes canted AFM.

Besides the magnetic properties of CPS, *H*_C_ or saturation fields (*H*_S_) of FGT also play a key role in exchange bias. For FGT with strong PMA,^[^ ^32^ ^]^ *H*_C_ is nearly the same as the *H*_S_. As shown in Figure 2f, thickness‐dependent *H*_C_ of FGT with three different thicknesses is plotted. *H*_C_ exhibits an apparent thickness dependence, i.e., the thinner of the thickness, the larger of the *H*_C_. The field required for the spinflop of CPS is relatively low, which is comparable to or even smaller than the *H*_C_ of FGT, especially for the thinner ones. Generally, to generate the unidirectional anisotropy and thus the exchange bias, the AFM/FM heterostructure is cooled down below the *T*_N_ with a *H*_CF_ a few times larger than the *H*_C_.^[^ ^33^ ^]^ For AFM/FM systems, a larger *H*_ex_ is expected when the FM sample is thinner while the AFM one is thicker, resulting from the enhanced proximity effect at the interface.^[^ ^14^ ^]^ Indeed, the *H*_ex_ of FGT(29.3 nm)/CPS(117.7 nm) is larger than that of FGT(52 nm)/CPS(120.2 nm) (Figure S6a, Supporting Information). However, for the 11.8 nm‐FGT‐based heterostructure, the *H*_C_ of FGT is extremely large (≈8700 Oe at 4 K). The energy barrier for this large *H*_C_ during the moments switching could overwhelm the unidirectional anisotropy, as is normally required for the effective exchange bias.^[^ ^13^, ^18^ ^]^ Consequently, the corresponding exchange bias of FGT(11.8 nm)/CPS(119.1 nm) is irregular and the magnitude of *H*_ex_ induced by PFC and NFC is distinct (Figure S6b, Supporting Information). In addition, given the required *H*_CF_ is already larger than the *H*_SF_, CPS has already undergone the spin‐flop transition. In this vein, a reliable and comprehensive field‐cooling‐dependent exchange bias research cannot be reached due to the large *H*_C_ of thin FGT and the low *H*_SF_ of CPS. Therefore, it is necessary to choose a suitable FM material with a relatively lower *H*_C_ to fully explore the modulation of the exchange bias via the spin‐flop transition.

**Figure 3:**
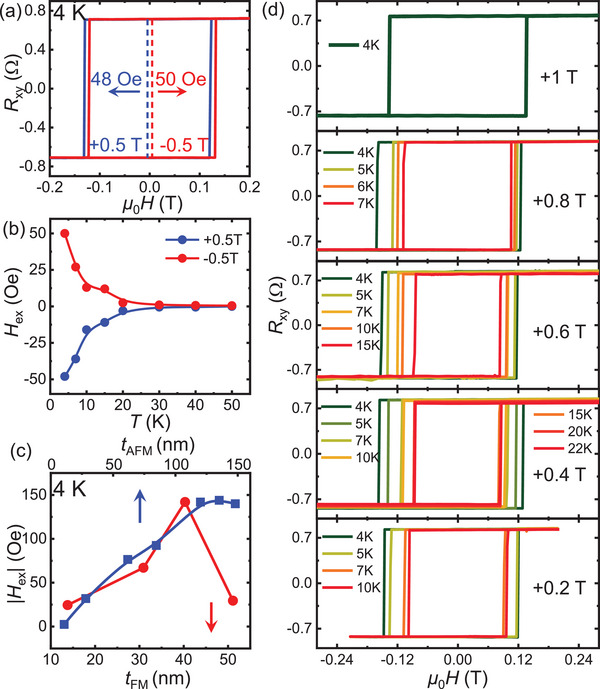
Thickness and field‐cooling dependence of the exchange bias effect in FCGT/CPS heterostructure. a) Field dependence of the Hall resistance *R*_xy_ measured at 4 K for FCGT(51.1 nm)/CPS(123.4 nm) heterostructure with applying the *H*_CF_ of +0.5 and −0.5 T, respectively. b) Temperature dependence of the |*H*_ex_| of FCGT(51.1 nm)/CPS(123.4 nm). c) Thickness dependence of the |*H*_ex_| at 4 K with the *H*_CF_ of +0.4 T of the FGT(≈40 nm)/CPS(*t*_AFM_) and FCGT(*t*_FM_)/CPS(≈120 nm). d) *H*_CF_‐dependent exchange bias effect of FCGT(40.3 nm)/CPS(119.2 nm) heterostructure. For clarity, we only plot the AHE loops for the temperature range of *T* ≤ *T*_B_ in each field cooling process.

According to recent work,^[^ ^34^, ^35^ ^]^ Co‐doped FGT shares the same crystal structure with FGT, but has a smaller *H*_C_, typically not exceeding 2000 Oe. This depressed *H*_C_ is due to decreased magnetic anisotropy with the Co dopant,^[^ ^35^, ^36^ ^]^ making FCGT suitable for investigating the influence of spin flop on exchange bias through the field‐cooling dependence. **Figure** **3**a shows Hall resistance of FCGT(51.1 nm)/CPS(123.4 nm) under the PFC and NFC of 0.5 T. Similar to the results of FGT/CPS (Figure 2a), negative exchange bias with |*H*_ex_| ≈50 Oe is observed. We note the smaller |*H*_ex_| compared with the one of FGT(52 nm)/CPS(120.2 nm) is ascribed to the depressed ferromagnetism of FCGT. The magnitude of the *H*_ex_ decreases with increasing temperature, and the *T*_B_ is ≈20 K, as shown in Figure 3b. Keeping CPS in the A‐type AFM state with a moderate *H*_CF_ of 0.4 T, we explore the exchange bias as a function of the thickness of both the FCGT (*t*_FM_) and the CPS (*t*_AFM_) (Figure 3c). When exploring the *t*_FM_‐dependent exchange bias, the *t*_AFM_ is fixed at ≈120 nm. While for the *t*_AFM_‐dependent one, the *t*_FM_ is fixed at ≈40 nm. It can be seen that the |*H*_ex_| of FGT(≈40 nm)/CPS(*t*_AFM_) increases from ≈2.5 to ≈140 Oe when the *t*_AFM_ increases from 10.7 to 120 nm, and the |*H*_ex_| saturates at ≈140 Oe with further increasing the *t*_AFM_. For the thickness of CPS below 10 nm, the exchange bias effect disappears due to the reduced anisotropy energy in CPS, which is not large enough to pin the spins in FGT during the magnetization reversal. Meanwhile, the |*H*_ex_| of FCGT(*t*_FM_)/CPS(≈120 nm) initially increases from 25 to 142 Oe as the *t*_FM_ increases from 13.8 to 40.3 nm and then decreases to 30 Oe when the *t*_FM_ is 51.1 nm, contrary to the conventional exchange bias of |*H*_ex_| ∝ 1/*t*_FM_ (i.e., |*H*_ex_| consistently decreases as increasing the *t*_FM_).^[^ ^16^ ^]^ This divergence between *H*_ex_ and 1/*t*_FM_ has also been observed in other vdW systems, such as FGT/CrCl_3_,^[^ ^13^ ^]^ FGT/Oxidized‐FGT,^[^ ^16^ ^]^ FGT/CoPc,^[^ ^14^ ^]^ and Fe_5_GeTe_2_/FePS_3_,^[^ ^18^ ^]^ possible due to the weak interlayer coupling^[^ ^16^ ^]^ or the lower volume magnetization of the thin FM flake cannot activate the AFM order sufficiently.^[^ ^14^ ^]^ Subsequently, we selected the FCGT(40.3 nm)/CPS(119.2 nm) device due to its largest *H*_ex_ among these heterostructures to investigate the cooling field dependence of exchange bias. Figure 3d summarizes the exchange bias under different field cooling processes, of which the *H*_CF_ can span the field required for the spin‐flop transition. We note that the *H*_ex_ and *T*_B_ are different for each field cooling process and we only plot the AHE loops for the temperature range of *T* ≤ *T*_B_ in Figure 3d. With the *H*_CF_ of +0.4 T, the shifted AHE loop disappears until 22 K. By contrast, under the *H*_CF_ of +1 T, the exchange bias nearly vanishes at 4 K. These differences determined by various *H*_CF_ imply that the spin‐flop transition indeed modulates the exchange bias effect.

**Figure 4:**
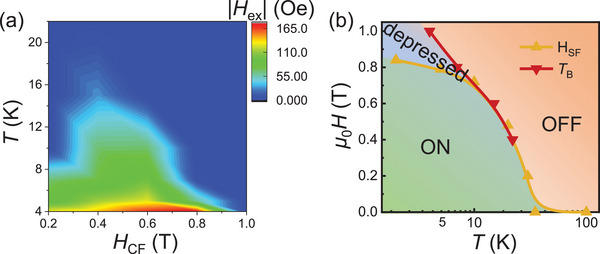
a) The magnitude of |*H*_ex_| of FCGT(40.3 nm)/CPS(119.2 nm) as a function of the temperature and cooling field, *H*_CF_. b) The modulation of the exchange bias in FCGT/CPS heterostructure through CPS spin‐flop transition.

To further analyze the modulation of spin flop on exchange bias, a contour map of the |*H*_ex_| as a function of temperature and *H*_CF_ is plotted in **Figure** **4**a. Note that when the *H*_CF_ = 0.2 T, the exchange bias of FCGT(40.3 nm)/CPS(119.2 nm) undergoes a rapid degradation as increasing the temperature and disappear at ≈10 K. These depressed *H*_ex_ and *T*_B_ under the *H*_CF_ of 0.2 T are predominately due to the relatively small *H*_CF_.^[^ ^33^ ^]^ Moreover, for the *H*_CF_ > 0.4 T, the area representing exchange bias shrinks in Figure 4a, indicating that *T*_B_ decreases with increasing the *H*_CF_. Specifically, *T*_B_ of 22 K is the largest one when *H*_CF_ = 0.4 T and decays upon increasing the *H*_CF_. This *H*_CF_‐modulated *T*_B_ is determined by the spin‐flop transition of CPS, which will be discussed later. Besides *T*_B_, we further explore the *H*_ex_ tailored by *H*_CF_, especially when *H*_CF_ surpasses the *H*_SF_. At a fixed temperature, it is obvious that the magnitude of *H*_ex_ does not increase and then saturates with the increasing *H*_CF_, as expected in FM/AFM systems.^[^ ^9^, ^15^ ^]^ However, |*H*_ex_| increases as the *H*_CF_ increases until the *H*_CF_ exceeds the *H*_SF,_ at which point the |*H*_ex_| starts to decrease. For instance, at 4 K, |*H*_ex_| increases from 130 to 177 Oe as the *H*_CF_ increases from 0.2 to 0.8 T. When the *H*_CF_ is larger than 0.8 T, at which field the spin‐flop transition of CPS occurs at 4 K, therefore, the CPS becomes a canted AFM. Correspondingly, magnetic moments begin to tilt and antiferromagnetically align perpendicular to the field. The uncompensated moments at the interfaces get weakened, thus the exchange bias effect is depressed. Further increasing the *H*_CF_ exceeding 1 T, moments of CPS have completely antiferromagnetically aligned in‐plane with small net moments along the *c* axis, suppressing the interfacial magnetic coupling as well as the exchange bias effect. During this spin‐flop process, the canted AFM phase reduces the interface magnetic coupling, leading to a degradation of the strength of the interfacial exchange coupling, *J*_FM‐AFM_, thereby suppressing the exchange bias (Figure S11, Supporting Information). Therefore, when the *H*_CF_ is larger than the *H*_SF_, the exchange bias effect is degraded or even washed out. Under this circumstance, if one applies a *H*_CF_ of 0.4 T, despite a rapid decrease of *H*_SF_ (from ≈0.8 to ≈0.4 T) in the temperature range of 10–20 K, *H*_SF_ is larger than the *H*_CF_ all along. CPS does not experience the spin flop and exchange bias is not influenced in the whole temperature range. Therefore, FCGT(40.3 nm)/CPS(119.2 nm) has the largest *T*_B_ when *H*_CF_ = 0.4 T. For the increased *H*_CF_ in other cases, *H*_CF_ generally goes beyond the *H*_SF_, especially at high temperatures. Exchange bias is depressed or eliminated at this temperature, thus leading to a lower *T*_B_ for the *H*_CF_ > 0.4 T. To sum up, the spin flop can effectively modulate the exchange bias of FCGT/CPS vdW heterostructure on both *T*_B_ and *H*_ex_.

Based on the temperature‐ and *H*_CF_‐ dependent *T*_B_ and *H*_SF_, the exchange bias effect modulated by spin‐flop transition is depicted in Figure 4b. When the *H*_CF_ is applied smaller than the *H*_SF_, the exchange bias is not influenced, displaying an “ON” state. However, once the conducted *H*_CF_ goes beyond the *H*_SF_, the spin‐flop transition begins where magnetic moments tilt and antiferromagnetically align perpendicular to the field. Consequently, exchange bias is depressed and the *T*_B_ is reduced, which is regarded as a “depressed” region. Moreover, further increasing the *H*_CF_ much exceeding the *H*_SF_, CPS has already become a canted AFM. In this vein, the interfacial magnetic coupling is eliminated and the exchange bias effect vanishes. It turns an “OFF” state. Therefore, by tuning the spin‐flop phase transition of CPS, we can select the desired exchange bias effect switching among the “ON”, “depressed”, and “OFF” states via setting up the appropriate cooling fields. Due to the fact that the AFM state of 2D vdW CPS has a relatively weak interlayer exchange coupling, it allows the low‐field switching between the A‐type AFM and canted states. It provides a perfect laboratory for studying exchange bias based on 2D antiferromagnetism. This spin‐flop modulated exchange bias effect has also been reproduced in different FCGT/CPS heterostructures with various CPS thicknesses (Figure S12, Supporting Information).

## 3 Conclusion

In summary, we have demonstrated the exchange bias effect modulated by AFM spin‐flop phase transition of CPS in 2D vdW magnetic heterostructures. Via AHE measurements, thickness‐ and cooling field‐dependent exchange bias effect is exhaustively investigated in both FGT/CPS and FCGT/CPS heterostructures. With the typical characteristic of CPS at a relatively low magnetic field, the spin‐flop transition can effectively depress the exchange bias of FCGT/CPS vdW heterostructure on both *T*_B_ and *H*_ex_. It allows one to select the desired exchange bias effect switching among the “ON”, “depressed”, and “OFF” states via conducting the appropriate cooling fields. This work provides an intrinsic approach, vi precisely setting up the magnetic phases of AFM materials to manipulate the exchange bias effect, showing great promise for spintronic devices based on vdW magnets.

## 4 Experimental Section

### Device Fabrication

Single‐crystal FGT, FCGT, and CPS were grown using the chemical vapor transport method, as previously.^[^ ^21^, ^23^ ^]^ Electrodes of Ti/Au (5 nm/25 nm) with standard Hall‐bar configuration were initially deposited on SiO_2_/Si substrates via electron beam lithography and electron beam evaporation. vdW heterostructures were fabricated using the dry transfer method. Thin flakes were mechanically exfoliated from the bulk using the scotch tape, and then suitable flake was picked up using PDMS. The thin layers of FGT or FCGT, followed by CPS, were sequentially transferred onto the pre‐patterned electrodes. Finally, the entire heterostructure was covered with a large‐area *h*‐BN to prevent oxidation and contamination during subsequent measurements. All device fabrications were conducted in a glovebox.

### Magnetism and Transport Measurements

Magnetic properties of CPS with an out‐of‐plane geometry were measured by the superconducting quantum interference device (SQUID). Transport properties were conducted via a cryogen‐free refrigeration system. Hall resistance and longitudinal resistance were measured simultaneously via a standard lock‐in technique with an applied current of 100 µA and a reference frequency of 13 Hz.

### Statistical Analysis

The Hall resistance in Figure 2b, Figures S3–S7 (Supporting Information) were offset for clarity.

## Conflict of Interest

The authors declare no conflict of interest.

## Supporting information

Supporting Information

## Data Availability

The data that support the findings of this study are available from the corresponding author upon reasonable request.

## References

[1] Q. H. Wang , A. Bedoya‐Pinto , M. Blei , A. H. Dismukes , A. Hamo , S. Jenkins , M. Koperski , Y. Liu , Q. C. Sun , E. J. Telford , H. H. Kim , M. Augustin , U. Vool , J. X. Yin , L. H. Li , A. Falin , C. R. Dean , F. Casanova , R. F. L. Evans , M. Chshiev , A. Mishchenko , C. Petrovic , R. He , L. Zhao , A. W. Tsen , B. D. Gerardot , M. Brotons‐Gisbert , Z. Guguchia , X. Roy , S. Tongay , et al., ACS Nano 2022, 16, 6960. 35442017 10.1021/acsnano.1c09150 PMC9134533

[2] E. M. Choi , K. I. Sim , K. S. Burch , Y. H. Lee , Adv. Sci. 2022, 9, 2200186. 10.1002/advs.202200186 PMC9313546 35596612

[3] X. Zhang , S. C. Ambhire , Q. Lu , W. Niu , J. Cook , J. S. Jiang , D. Hong , L. Alahmed , L. He , R. Zhang , Y. Xu , S. S.‐L. Zhang , P. Li , G. Bian , ACS Nano 2021, 15, 15710. 34460216 10.1021/acsnano.1c05519

[4] Y. Chen , Y. Zhu , R. Lin , W. Niu , R. Liu , W. Zhuang , X. Zhang , J. Liang , W. Sun , Z. Chen , Y. Hu , F. Song , J. Zhou , D. Wu , B. Ge , H. Yang , R. Zhang , X. Wang , Adv. Funct. Mater. 2023, 33, 2302984.

[5] H. Dai , M. Cai , Q. Hao , Q. Liu , Y. Xing , H. Chen , X. Chen , X. Wang , H.‐H. Fu , J. Han , ACS Nano 2022, 16, 12437. 35900014 10.1021/acsnano.2c03626

[6] J. F. Sierra , J. Fabian , R. K. Kawakami , S. Roche , S. O. Valenzuela , Nat. Nanotechnol. 2021, 16, 856. 34282312 10.1038/s41565-021-00936-x

[7] Y. Hou , J. Kim , R. Wu , Sci. Adv. 2019, 5, eaaw1874. 31172028 10.1126/sciadv.aaw1874 PMC6544448

[8] S. Liu , X. Yuan , Y. Zou , Y. Sheng , C. Huang , E. Zhang , J. Ling , Y. Liu , W. Wang , C. Zhang , J. Zou , K. Wang , F. Xiu , npj 2D Mater. Appl. 2017, 1, 30.

[9] G. Hu , Y. Zhu , J. Xiang , T. Y. Yang , M. Huang , Z. Wang , Z. Wang , P. Liu , Y. Zhang , C. Feng , D. Hou , W. Zhu , M. Gu , C. H. Hsu , F. C. Chuang , Y. Lu , B. Xiang , Y. L. Chueh , ACS Nano 2020, 14, 12037. 32885948 10.1021/acsnano.0c05252

[10] H. Dai , H. Cheng , M. Cai , Q. Hao , Y. Xing , H. Chen , X. Chen , X. Wang , J. B. Han , ACS Appl. Mater. Interfaces 2021, 13, 24314. 33977712 10.1021/acsami.1c05265

[11] L. Zhang , X. Huang , H. Dai , M. Wang , H. Cheng , L. Tong , Z. Li , X. Han , X. Wang , L. Ye , J. Han , Adv. Mater. 2020, 32, 2002032. 10.1002/adma.202002032 32803805

[12] X. Huang , L. Zhang , L. Tong , Z. Li , Z. Peng , R. Lin , W. Shi , K.‐H. Xue , H. Dai , H. Cheng , D. de Camargo Branco , J. Xu , J. Han , G. J. Cheng , X. Miao , L. Ye , Nat. Commun. 2023, 14, 2190. 37069179 10.1038/s41467-023-37918-7 PMC10110563

[13] R. Zhu , W. Zhang , W. Shen , P. K. J. Wong , Q. Wang , Q. Liang , Z. Tian , Y. Zhai , C. W. Qiu , A. T. S. Wee , Nano Lett. 2020, 20, 5030. 32463247 10.1021/acs.nanolett.0c01149

[14] J. Jo , F. Calavalle , B. Martin‐Garcia , D. Tezze , F. Casanova , A. Chuvilin , L. E. Hueso , M. Gobbi , Adv. Mater. 2022, 34, 2200474. 10.1002/adma.202200474 35334502

[15] T. Zhang , Y. Zhang , M. Huang , B. Li , Y. Sun , Z. Qu , X. Duan , C. Jiang , S. Yang , Adv. Sci. 2022, 9, 2105483. 10.1002/advs.202105483 PMC9009105 35238180

[16] H. K. Gweon , S. Y. Lee , H. Y. Kwon , J. Jeong , H. J. Chang , K. W. Kim , Z. Q. Qiu , H. Ryu , C. Jang , J. W. Choi , Nano Lett. 2021, 21, 1672. 33570963 10.1021/acs.nanolett.0c04434

[17] Q. Wu , Y. Zhang , Z. Cui , P. Liu , B. Xiang , Z. Li , Z. Fu , Y. Lu , Adv. Funct. Mater. 2023, 33, 2214007.

[18] S. Albarakati , W. Q. Xie , C. Tan , G. Zheng , M. Algarni , J. Li , J. Partridge , M. J. S. Spencer , L. Farrar , Y. Xiong , M. Tian , X. Wang , Y. J. Zhao , L. Wang , Nano Lett. 2022, 22, 6166. 35912475 10.1021/acs.nanolett.2c01370

[19] J.‐Z. Fang , H.‐N. Cui , S. Wang , J.‐D. Lu , G.‐Y. Zhu , X.‐J. Liu , M.‐S. Qin , J.‐K. Wang , Z.‐N. Wu , Y.‐F. Wu , S.‐G. Wang , Z.‐S. Zhang , Z. Wei , J. Zhang , B.‐C. Lin , Z.‐M. Liao , D. Yu , Phys. Rev. B 2023, 107, L041107.

[20] Z. Ying , B. Chen , C. Li , B. Wei , Z. Dai , F. Guo , D. Pan , H. Zhang , D. Wu , X. Wang , S. Zhang , F. Fei , F. Song , Nano Lett. 2023, 23, 765. 36542799 10.1021/acs.nanolett.2c02882

[21] W. Niu , Z. Cao , Y. Wang , Z. Wu , X. Zhang , W. Han , L. Wei , L. Wang , Y. Xu , Y. Zou , L. He , Y. Pu , Phys. Rev. B 2021, 104, 125429.

[22] X. Chen , H. Wang , M. Li , Q. Hao , M. Cai , H. Dai , H. Chen , Y. Xing , J. Liu , X. Wang , T. Zhai , X. Zhou , J. B. Han , Adv. Sci. 2023, 10, 2207617. 10.1002/advs.202207617 PMC10401167 37327250

[23] W. Li , Y. Dai , L. Ni , B. Zhang , D. Tang , Y. Yang , Y. Xu , Adv. Funct. Mater. 2023, 33, 2303781.

[24] Y. Peng , S. Ding , M. Cheng , Q. Hu , J. Yang , F. Wang , M. Xue , Z. Liu , Z. Lin , M. Avdeev , Y. Hou , W. Yang , Y. Zheng , J. Yang , Adv. Mater. 2020, 32, 2001200. 10.1002/adma.202001200 32500563

[25] F. Wu , M. Gibertini , K. Watanabe , T. Taniguchi , I. Gutiérrez‐Lezama , N. Ubrig , A. F. Morpurgo , Adv. Mater. 2023, 35, 2211653. 10.1002/adma.202211653 37098224

[26] S. Ding , Y. Peng , M. Xue , Z. Liu , Z. Liang , W. Yang , Y. Sun , J. Zhao , C. Wang , S. Liu , J. Han , J. Yang , J. Phys.:Condens. Matter 2020, 32, 405804. 32554867 10.1088/1361-648X/ab9e2d

[27] G. Zheng , W. Q. Xie , S. Albarakati , M. Algarni , C. Tan , Y. Wang , J. Peng , J. Partridge , L. Farrar , J. Yi , Y. Xiong , M. Tian , Y. J. Zhao , L. Wang , Phys. Rev. Lett. 2020, 125, 047202. 32794802 10.1103/PhysRevLett.125.047202

[28] C. Liu , H. Zhang , S. Zhang , D. Hou , Y. Liu , H. Wu , Z. Jiang , H. Wang , Z. Ma , X. Luo , X. Li , Y. Sun , X. Xu , Z. Zhang , Z. Sheng , Adv. Mater. 2023, 35, 2203411. 10.1002/adma.202203411 36300686

[29] J. Liang , S. Liang , T. Xie , A. F. May , T. Ersevim , Q. Wang , H. Ahn , C. Lee , X. Zhang , J.‐P. Wang , M. A. McGuire , M. Ouyang , C. Gong , Phys. Rev. Mater. 2023, 7, 014008.

[30] B. Huang , G. Clark , E. Navarro‐Moratalla , D. R. Klein , R. Cheng , K. L. Seyler , D. Zhong , E. Schmidgall , M. A. McGuire , D. H. Cobden , W. Yao , D. Xiao , P. Jarillo‐Herrero , X. Xu , Nature 2017, 546, 270. 28593970 10.1038/nature22391

[31] J. Son , S. Son , P. Park , M. Kim , Z. Tao , J. Oh , T. Lee , S. Lee , J. Kim , K. Zhang , K. Cho , T. Kamiyama , J. H. Lee , K. F. Mak , J. Shan , M. Kim , J.‐G. Park , J. Lee , ACS Nano 2021, 15, 16904. 34661389 10.1021/acsnano.1c07860

[32] W. Niu , X. Zhang , W. Wang , J. Sun , Y. Xu , L. He , W. Liu , Y. Pu , Appl. Phys. Lett. 2021, 119, 172402.

[33] P. Miltényi , M. Gierlings , M. Bamming , U. May , G. Güntherodt , J. Nogués , M. Gruyters , C. Leighton , I. K. Schuller , Appl. Phys. Lett. 1999, 75, 2304.

[34] R. R. Chowdhury , S. DuttaGupta , C. Patra , O. A. Tretiakov , S. Sharma , S. Fukami , H. Ohno , R. P. Singh , Sci. Rep. 2021, 11, 14121. 34238967 10.1038/s41598-021-93402-6 PMC8266818

[35] Z. Wu , W. Niu , W. Li , J. Yang , K. Gu , X. Liu , X. Wang , S. Chang , L. Wei , F. Li , P. Liu , X. Zhang , J. Ma , L. He , Y. Xu , Y. Pu , Appl. Phys. Lett. 2023, 123, 192403.

[36] G. Drachuck , Z. Salman , M. W. Masters , V. Taufour , T. N. Lamichhane , Q. Lin , W. E. Straszheim , S. L. Bud'ko , P. C. Canfield , Phys. Rev. B 2018, 98, 144434.

