# Supplementary material for: Exchange Bias Modulated by Antiferromagnetic Spin‐Flop Transition in 2D Van der Waals Heterostructures
Source: Adv Sci (Weinh). 2024 Feb 14;11(17):2307034. doi: 10.1002/advs.202307034 (PMC11077673; doi:10.1002/advs.202307034)
Supplement: Supplementary file 1 — Supporting Information [file ADVS-11-2307034-s001.pdf]

ADVANCED SCIENCE
Open Access

Supporting Information

for *Adv. Sci.*, DOI 10.1002/advs.202307034

Exchange Bias Modulated by Antiferromagnetic Spin-Flop Transition in 2D Van der Waals Heterostructures

Kai Gu, Xiaoqian Zhang, Xiangjie Liu, Xinlei Guo, Zhenqi Wu, Shuo Wang, Qinxin Song, Wei Wang, Lujun Wei, Ping Liu, Jingrui Ma, Yongbing Xu, Wei Niu and Yong Pu**

Supporting Information

Exchange Bias Modulated by Antiferromagnetic Spin-Flop Transition in Two-Dimensional van der Waals Heterostructures

Kai Gu, Xiaoqian Zhang, Xiangjie Liu, Xinlei Guo, Zhenqi Wu, Shuo Wang, Qinxin Song, Wei Wang, Lujun Wei, Ping Liu, Jingrui Ma, Yongbing Xu, Wei Niu*, and Yong Pu*

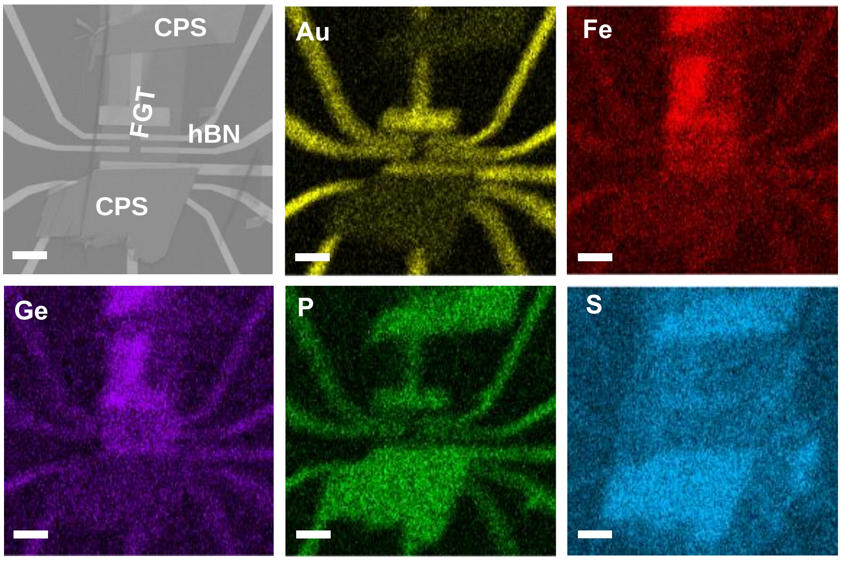

Figure S1. Scanning electron microscopy image of FGT/CPS heterostructure and the corresponding energy-dispersive X-ray spectroscopy (EDX) mappings of Au, Fe, Ge, P, and S elements, respectively. The scale bar is 10 μm. Within the resolution, EDX results demonstrate a clear distribution of different elements in corresponding regions, with well-defined boundaries between FGT and CPS.

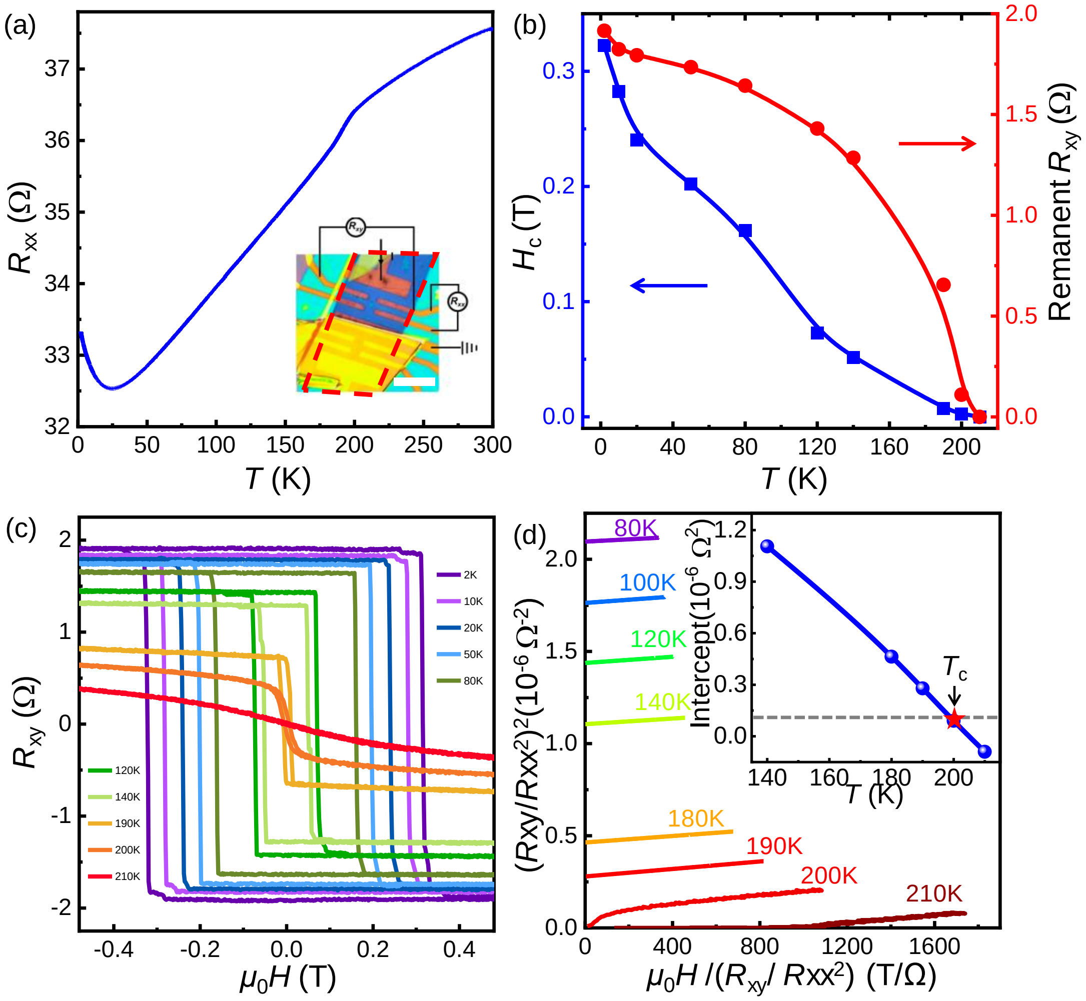

Figure S2. Transport properties of pure FGT measured without field cooling. (a) Longitudinal resistance (R_{xx}) as a function of temperature. The inset depicts the measurement geometry for the R_{xx} and Hall resistance, R_{xy}. (b) Temperature dependence of the coercivity (H_C) and remanent R_{xy}. (c) Magnetic field dependence of R_{xy} at different temperatures. No exchange bias is observed in the pure FGT region. (d) Arrott-plot analyses of FGT at different temperature near the T_C. The inset shows a liner fit of the intercept, signifying the T_C of ~ 200 K of the FGT nanodevice.

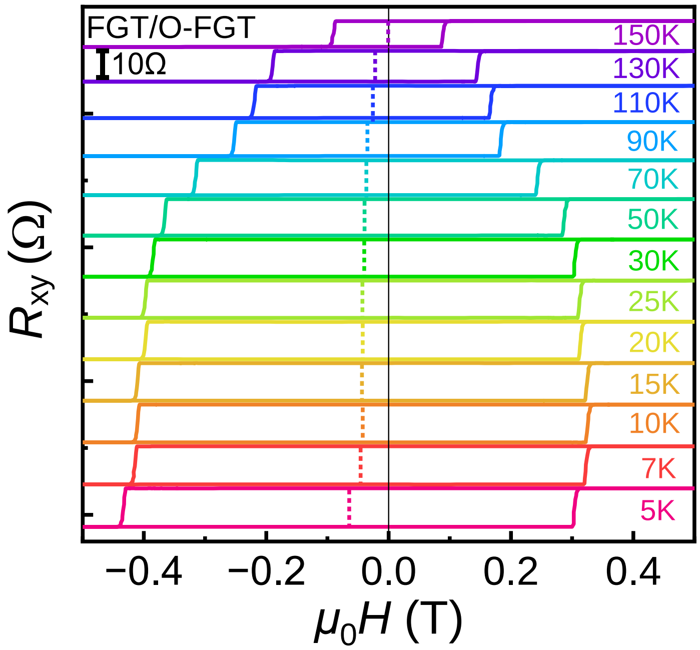

Figure S3. Field-dependent R_{xy} of Fe_3GeTe_2 with surface oxidization at different temperatures. Exchange bias disappears at 150 K, signifying the blocking temperature of 150 K.

Recently, exchange bias has been observed in Fe_3GeTe_2 with surface oxidization due to the antiferromagnetic characteristic of the oxidized Fe_3GeTe_2.[1-4] We tested a Fe_3GeTe_2 flake (~ 40 nm) intentionally exposed to air 30 min without any encapsulation film. Indeed, as displayed in Figure S3, exchange bias is observed in Fe_3GeTe_2 with surface oxidization. Notably, the blocking temperature, T_B, of oxidized-Fe_3GeTe_2/Fe_3GeTe_2 (~ 150 K) is much higher than both the one of Fe_3GeTe_2/$CrPS_4$ and the Néel temperature of $CrPS_4$. Although the exchange bias effect is achieved in Fe_3GeTe_2 with surface oxidation, the intrinsic magnetic couplings between Fe_3GeTe_2 and $CrPS_4$ are more desired in this work. In this vein, surface oxidation should be avoided. Therefore, our samples were encapsulated by a large-area *h*-BN and fabricated in a glovebox to prevent the oxidization-induced exchange bias.

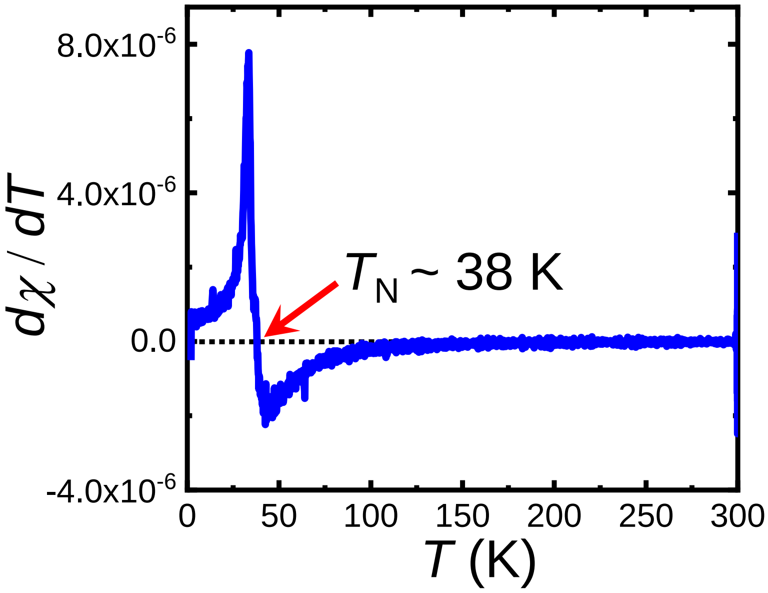

Figure S4. Temperature-dependent derivative of the susceptibility ($d\chi/dT$) of $CrPS_4$. It crosses zero at around 38 K, further indicating the T_N of ~ 38 K.

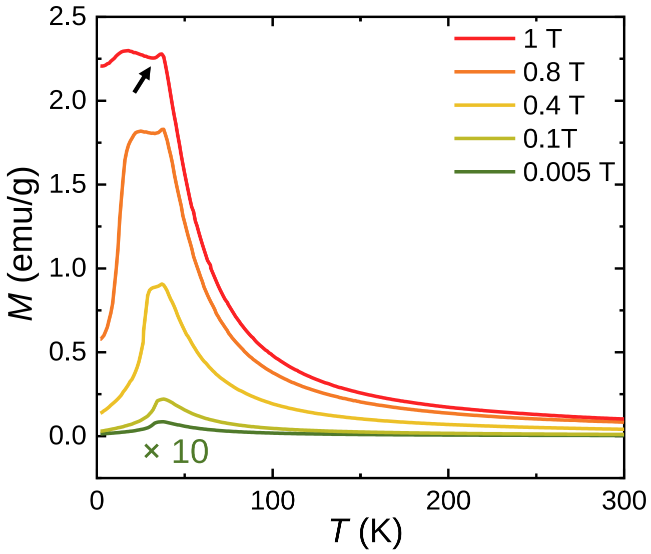

Figure S5. Temperature-dependent magnetization (*M*-*T*) curves of $CrPS_4$ under various applied fields.

As shown in Figure S5, under a low magnetic field of 0.005 T (CPS is at the A-type AFM state), the *M*-*T* curve displays a typical AFM behavior, where the magnetization increases as temperature decreases and reaches a maximum at 38 K, and then it begins to decrease dramatically. This AFM transition corresponds to AFM order originating from exchange coupling between Cr atoms in the neighboring sublattices and displays a T_N of 38 K, which is in good consistence with previous reports.[5, 6] Meanwhile, by adopting the magnetic field of 1 T, CPS is in the canted AFM regime. Under this circumstance, the magnetization decreases slowly and faintly upon cooling when the temperature is below T_N. It is obvious that the low-temperature (2 K) magnetization at magnetic field of 1T is much larger than that of 0.005 T, which is due to the contribution of the canted AFM state. We note that there is an abnormal minimum (marked by black arrow) below T_N in the *M*-*T* curve, which is induced by short-range ferromagnetic correlations, competing with AFM ordering.[5, 7]

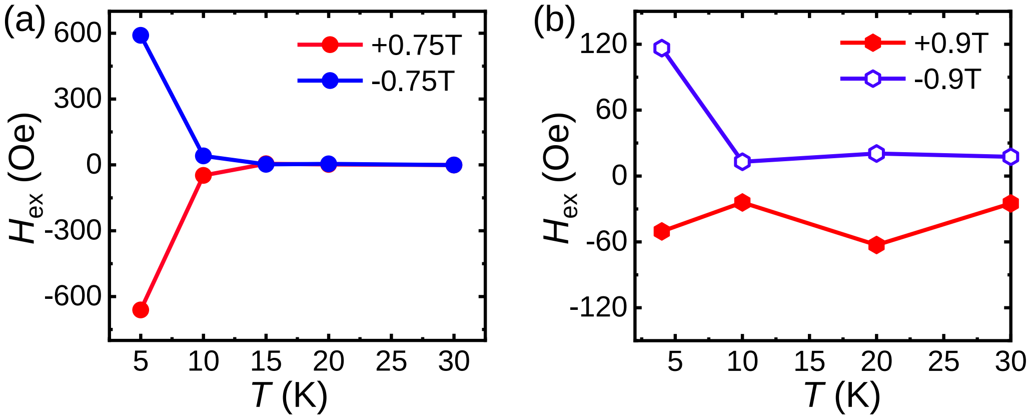

Figure S6. Exchange bias effect in FGT(29.3 nm)/CPS(117.7 nm) and FGT(11.8 nm)/CPS(119.1 nm). (a) Temperature-dependent H_{ex} of FGT(29.3 nm)/CPS(117.7 nm). $|H_{ex}|$ decreases with increasing the temperature with a T_B of 15 K under the PFC of +0.75 T and the NFC of -0.75 T, respectively. (b) Temperature-dependent H_{ex} of FGT(11.8 nm)/CPS (119.1 nm). The magnitude of H_{ex} in FGT(11.8 nm)/CPS (119.1 nm) under the PFC and the NFC is not identical. It should be noted that the adopted cooling fields are different for the FGT(29.3 nm)/CPS(117.7 nm) and FGT(11.8 nm)/CPS(119.1 nm), because the coercive fields of these two FGT flakes are different. Generally, to generate the unidirectional anisotropy and thus the exchange bias, the AFM/FM heterostructure is cooled down below the Néel temperature with a cooling field larger than the coercivity.

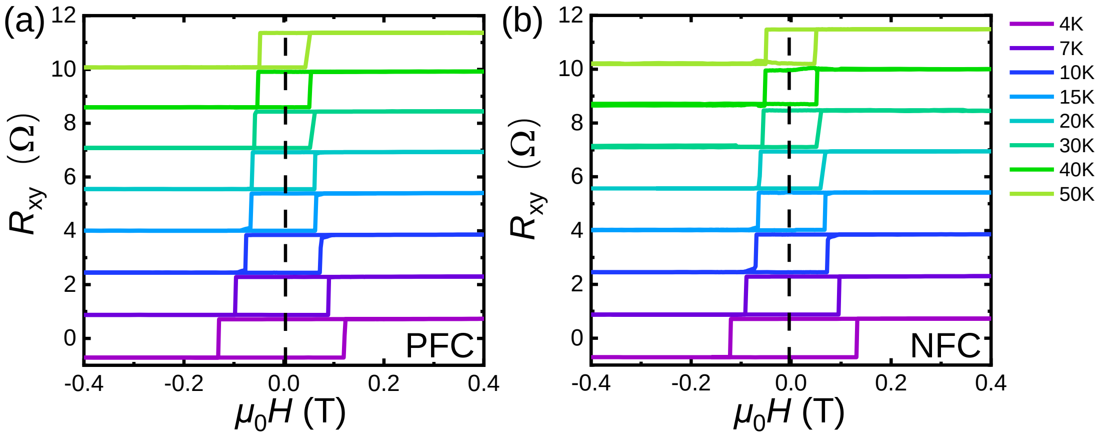

Figure S7. Field dependence of the R_{xy} measured at different temperatures for FCGT (51.1 nm)/CPS(123.4 nm) heterostructure with (a) PFC of +0.5 T and (b) NFC of -0.5 T, respectively.

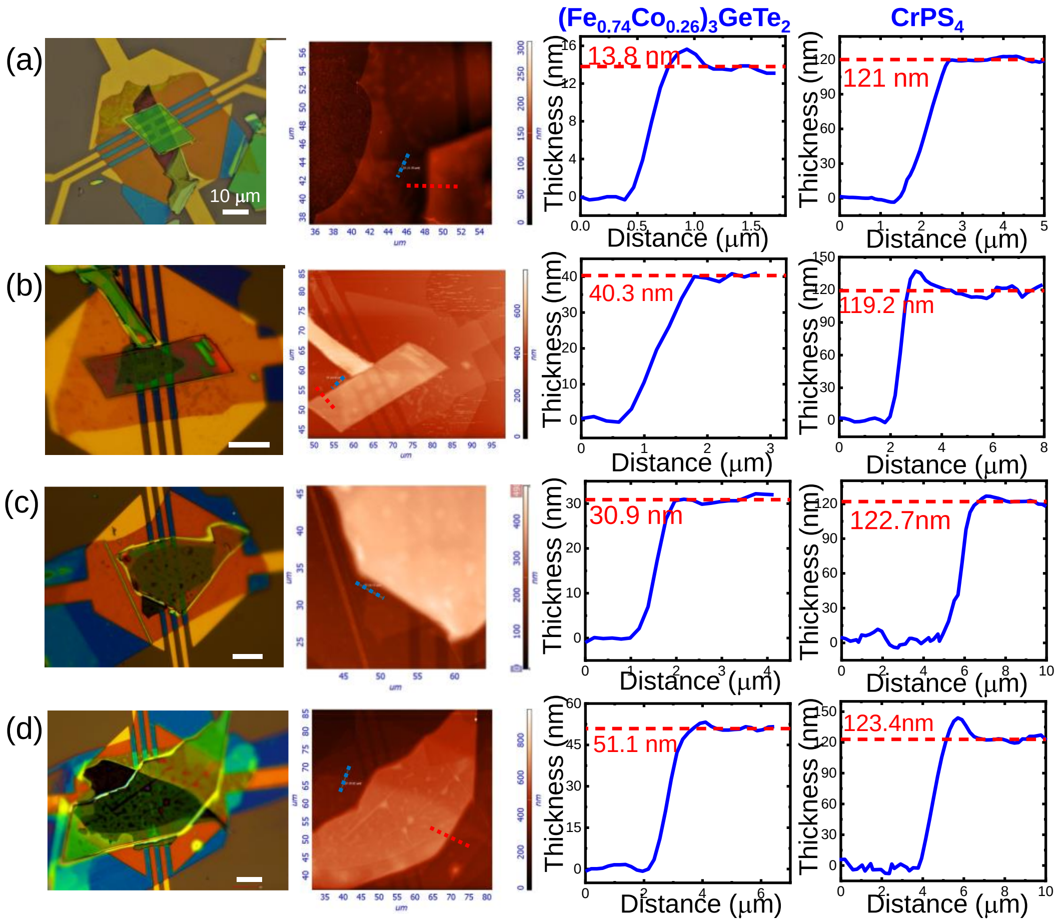

Figure S8. Optical, atomic force microscopy images, and corresponding height profiles of four FCGT/CPS heterostructures with different thickness of FCGT. The thicknesses of CPS flakes are around 120 nm.

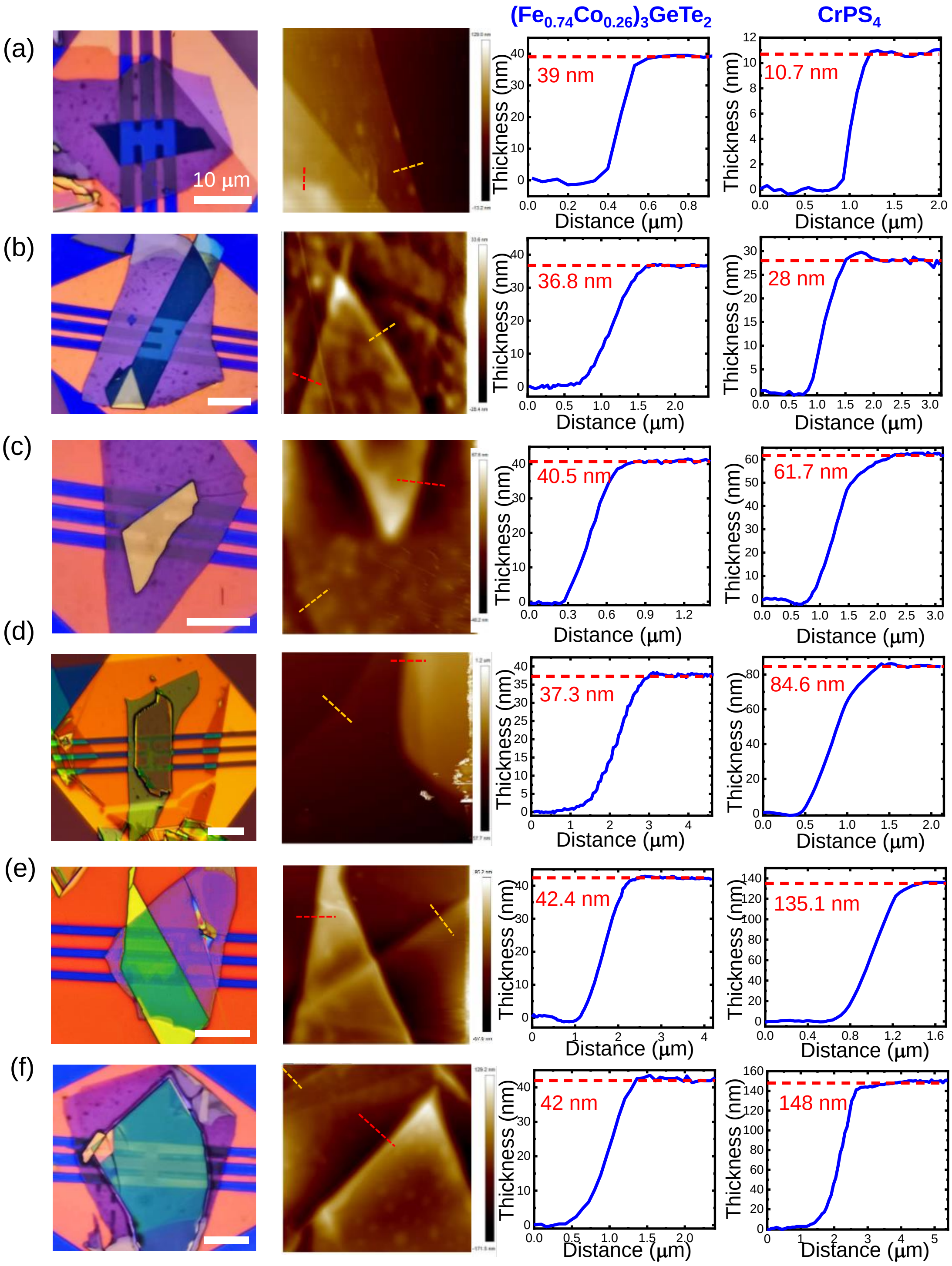

Figure S9. Optical, atomic force microscopy images and corresponding height profiles of the six FCGT/CPS heterostructures with different CPS thickness. The thicknesses of FCGT in these devices are around 40 nm.

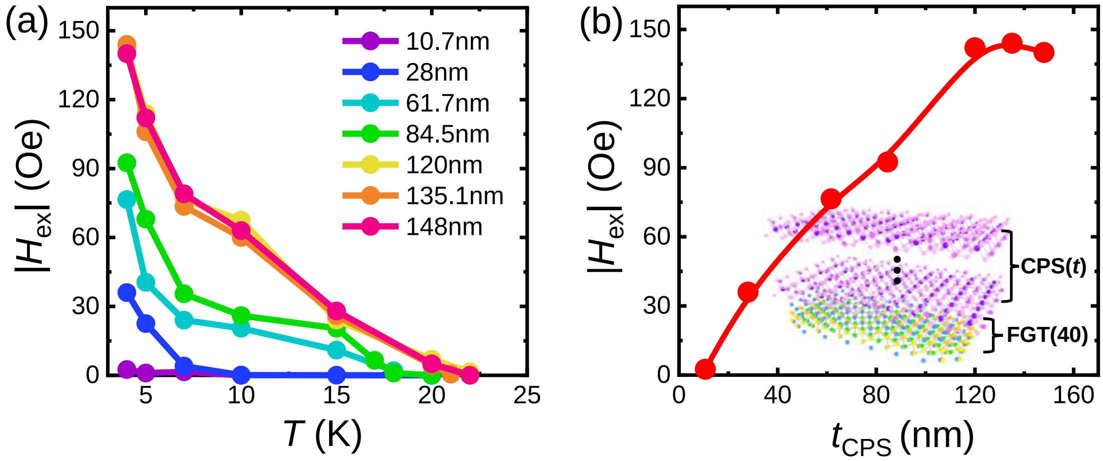

Figure S10. CPS thickness-dependent exchange bias in FCGT(~40 nm)/CPS heterostructures. (a) Temperature dependence of the $|H_{ex}|$ with different CPS thicknesses under a cooling field of 0.4 T. (b) $|H_{ex}|$ at 4 K as a function of the CPS thickness.

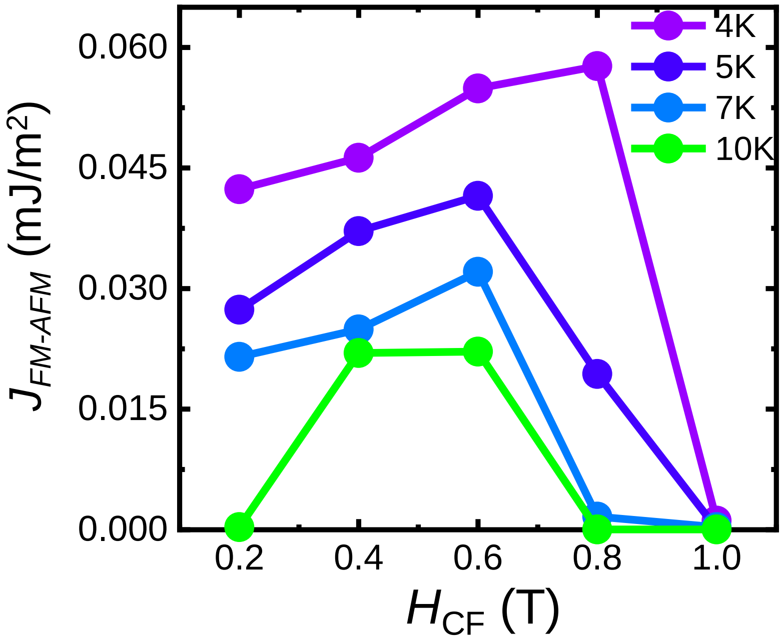

Figure S11. Cooling field dependence of $J_{\mathrm{FM\text{-}AFM}}$ for the exchange bias effect in FCGT(40.3 nm)/CPS(119.2 nm) heterostructure at different temperatures.

According to the Meiklejohn–Bean model,[8] exchange bias field, H_{ex}, is calculated as:

$$H_{\mathrm{ex}} = -\frac{J_{\mathrm{FM\text{-}AFM}}}{\mu_0 M_{\mathrm{FM}} t_{\mathrm{FM}}}$$

where μ_0 is the vacuum permeability, $J_{\mathrm{FM\text{-}AFM}}$ is the strength of the interface exchange coupling between FM layer and AFM layer, M_{FM} is the saturation magnetization of the FM layer per unit volume, and t_{FM} is the thickness of FM layer. During the measurement, the M_{FM} and t_{FM} remain unchanged at a fixed temperature. Therefore, the exchange bias field is determined by the $J_{\mathrm{FM\text{-}AFM}}$. Hence, the spin-flop transition alters the exchange bias field by influencing the strength of the interface exchange coupling $J_{\mathrm{FM\text{-}AFM}}$. The value of M_{FM}, approximately 80 emu/cm^3, is obtained from SQUID data of intrinsic FCGT bulk material. When the applied field cooling is sufficient to induce a spin-flop transition in CPS, there is a noticeable decrease in $J_{\mathrm{FM\text{-}AFM}}$, leading to the reduction of the H_{ex} and the degradation of the exchange bias.

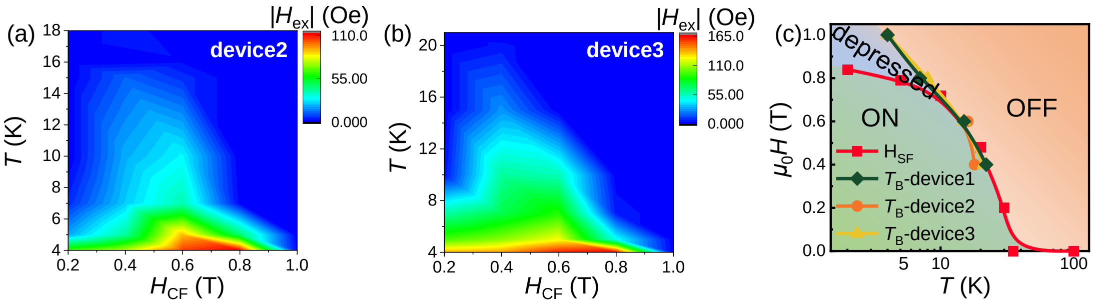

Figure S12. The magnitude of exchange bias field, $|H_{ex}|$, as a function of the temperature and cooling field, H_{CF}, of different FCGT/CPS heterostructures. (a) FCGT(40.5 nm)/CPS(61.7 nm), i.e., device 2. (b) FCGT(42 nm)/CPS(148 nm), i.e., device 3. (c) The modulation of the exchange bias through CPS spin-flop transition in three different FCGT/CPS heterostructures. Note that device 1 is the heterostructure of FCGT(40.3 nm)/CPS(119.2 nm) shown in the main text. These results indicate that spin-flop transition modulated exchange bias effect can be reproduced in other heterostructures with different CPS thickness.

Reference:

[1] Q. Wu, Y. Zhang, Z. Cui, P. Liu, B. Xiang, Z. Li, Z. Fu, Y. Lu, *Adv. Funct. Mater.* **2023**, 33, 2214007.

[2] J. Liang, S. Liang, T. Xie, A. F. May, T. Ersevim, Q. Wang, H. Ahn, C. Lee, X. Zhang, J.-P. Wang, M. A. McGuire, M. Ouyang, C. Gong, *Phys. Rev. Mater.* **2023**, 7, 014008.

[3] S. Hu, X. Cui, Z. Yue, P. Wang, L. Guo, K. Ohnishi, X. Wang, T. Kimura, *2D Mater.* **2022**, 9, 015037.

[4] H. K. Gweon, S. Y. Lee, H. Y. Kwon, J. Jeong, H. J. Chang, K. W. Kim, Z. Q. Qiu, H. Ryu, C. Jang, J. W. Choi, *Nano Lett.* **2021**, 21, 1672.

[5] Y. Peng, S. Ding, M. Cheng, Q. Hu, J. Yang, F. Wang, M. Xue, Z. Liu, Z. Lin, M. Avdeev, Y. Hou, W. Yang, Y. Zheng, J. Yang, *Adv. Mater.* **2020**, 32, 2001200.

[6] J. Son, S. Son, P. Park, M. Kim, Z. Tao, J. Oh, T. Lee, S. Lee, J. Kim, K. Zhang, K. Cho, T. Kamiyama, J. H. Lee, K. F. Mak, J. Shan, M. Kim, J.-G. Park, J. Lee, *ACS Nano* **2021**, 15, 16904.

[7] M. A. McGuire, G. Clark, S. Kc, W. M. Chance, G. E. Jellison, V. R. Cooper, X. Xu, B. C. Sales, *Phys. Rev. Mater.* **2017**, 1, 014001.

[8] W. H. Meiklejohn, C. P. Bean, *Physical review* **1956**, 102, 1413.
